# Primary Syphilis Presenting as an Atypical Facial Chancre: Diagnostic Value of Extraoral Examination in Dental Practice

**DOI:** 10.7759/cureus.111134

**Published:** 2026-06-19

**Authors:** Ziad Farih, Nadia El Wadidi, Youssef Naji

**Affiliations:** 1 Department of Oral Surgery, Mohammed VI University of Health and Medical Sciences, Casablanca, MAR

**Keywords:** chancre, dental examination, early diagnosis, physical examination, primary, syphilis

## Abstract

Syphilis, a chronic sexually transmitted infection caused by *Treponema pallidum*, is reemerging globally with increasingly diverse clinical presentations. Extragenital lesions, particularly on the face, may be misdiagnosed or overlooked during routine medical or dental examinations. We report the incidental diagnosis of primary syphilis in a 43-year-old male patient who presented for a routine dental evaluation. During extraoral examination, an asymptomatic, indurated ulcer on the left frontal region was discovered. Serological testing confirmed the diagnosis with positive Venereal Disease Research Laboratory (VDRL; 1:2) and *Treponema pallidum* hemagglutination assay (TPHA; 217.9) results. This case illustrates the crucial role of dental practitioners in recognizing systemic diseases through comprehensive extraoral assessment. Although primarily focused on oral health, dentists are often the first point of contact for patients and thus uniquely positioned to detect early manifestations of infectious conditions such as syphilis. A systematic extraoral examination should be an integral part of every dental visit. Early identification of atypical lesions not only facilitates timely medical referral but also enhances the dentist’s contribution to global public health surveillance.

## Introduction

Syphilis, a sexually transmitted infection caused by *Treponema pallidum*, has experienced a notable resurgence globally in recent years. Although its primary manifestations are typically genital, syphilis is renowned as “the great imitator” due to its diverse clinical presentations, which can involve the skin, mucosa, or other organs. A chancre is the characteristic lesion of primary syphilis, classically presenting as a solitary, painless, indurated ulcer at the site of inoculation. Because inoculation most commonly occurs on genital, oral, or perioral mucosal surfaces, a lesion located on the forehead represents an unusual extragenital presentation and may easily be mistaken for traumatic, infectious, or neoplastic skin disease. Cutaneous lesions, particularly in the primary stage, may be overlooked or misdiagnosed when they appear in uncommon locations [1]. While dentists are primarily focused on the oral cavity, a thorough extraoral examination remains an essential part of the clinical assessment. In some cases, this broader examination allows dental professionals to detect signs suggestive of systemic conditions that may otherwise go unnoticed. Recent reports have underlined the critical role dentists can play in the early identification of infectious diseases, including syphilis, through incidental findings during routine evaluations [2]. We present the case of a patient who sought only dental care, but whose extraoral examination revealed a chancre-like lesion on the forehead. This observation led to serological testing and the diagnosis of primary syphilis. This case underscores the value of comprehensive clinical assessment in dental practice and the role of oral health professionals in the early detection of systemic infections.

## Case presentation

Patient information

A 43-year-old married male patient, born on November 2, 1979, presented to our department for a routine dental evaluation. He reported no systemic complaints and had no history of significant medical or surgical conditions. The consultation was not prompted by any pain or discomfort.

Clinical findings

**Figure 1:**
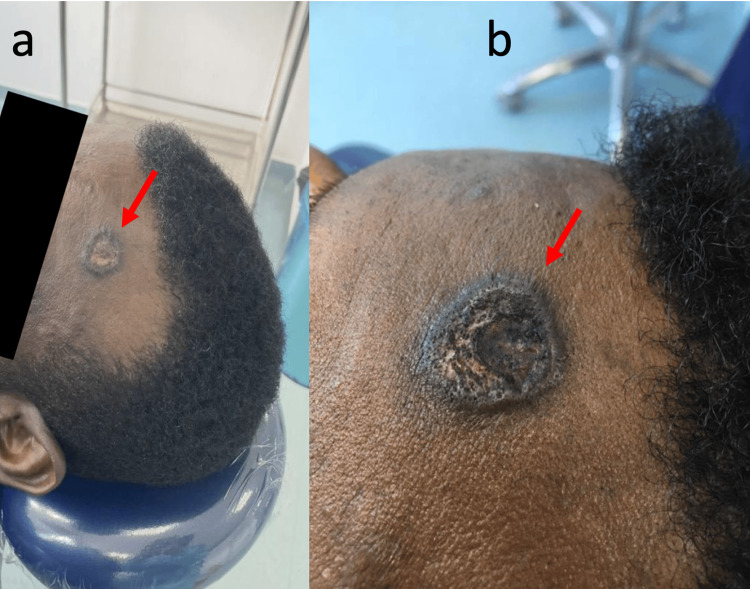
Extraoral clinical presentation of the lesion. a: General view showing the lesion on the left frontal region. b: Close-up view demonstrating a solitary, well-defined, indurated ulcerative lesion with a crusted surface. The lesion was asymptomatic and was incidentally identified during extraoral examination, leading to the diagnosis of primary syphilis.

During the extraoral examination, a solitary ulcerated lesion measuring approximately 2 cm in diameter was observed on the left frontal region. The lesion had a clean, indurated base with well-defined borders and was non-tender to palpation (Figure 1). On further questioning, the patient reported that the lesion had been present for several weeks. He reported no associated symptoms, and a subsequent genital examination performed during specialist medical evaluation did not identify any genital lesion. Palpation of the regional lymph node areas, including the cervical, submandibular, preauricular, and occipital regions, revealed no palpable lymphadenopathy. Intraoral examination was unremarkable, with no mucosal or periodontal lesions.

Timeline

The chronological sequence of clinical findings, diagnostic workup, treatment, and follow-up is summarized in Table 1.

**Table 1:** Clinical timeline of diagnosis, treatment, and follow-up. VDRL, Venereal Disease Research Laboratory test; TPHA: Treponema pallidum hemagglutination assay.

Time point	Clinical event
Initial dental consultation	The patient presented for a routine dental evaluation without pain or systemic complaints.
Same visit	Extraoral examination revealed an asymptomatic, indurated ulcerative lesion on the left frontal region.
Shortly after clinical suspicion	Serological testing was requested, showing positive VDRL at 1:2 dilution and positive TPHA at 217.9.
After diagnosis confirmation	The patient was referred to his general practitioner and received benzathine penicillin G 2.4 million units as a single intramuscular dose.
Follow-up at 6 months	Repeat VDRL testing was negative, and clinical examination showed complete resolution of the frontal lesion.

Diagnostic assessment

Given the clinical appearance of the lesion, a syphilitic chancre was suspected. Serological tests were prescribed to investigate this possibility. The Venereal Disease Research Laboratory (VDRL) test returned positive at 1:2 dilution, and the *Treponema pallidum* hemagglutination assay (TPHA) was markedly elevated at 217.9 (reference value: <1), confirming the diagnosis of primary syphilis (Table 2). Other differential diagnoses considered included a traumatic ulcer, cutaneous leishmaniasis, and a non-healing skin neoplasm, but these were excluded based on the clinical context, lesion characteristics, and the serological confirmation of treponemal infection.

**Table 2:** Serological test results confirming the diagnosis of primary syphilis. VDRL, Venereal Disease Research Laboratory test; TPHA: *Treponema pallidum* hemagglutination assay.

Diagnostic test	Result	Normal range	Interpretation
VDRL	1:2	Negative	Positive
TPHA	217.9	<1	Positive

Therapeutic intervention

Due to logistical constraints, treatment and follow-up were conducted in coordination with the patient's general practitioner. Based on the diagnosis, the patient was referred to a general practitioner for management. Antibiotic therapy was initiated according to current guidelines, namely benzathine penicillin G (BPG) 2.4 MU administered as a single intramuscular dose.

Follow-up and outcomes

Six months after the initial positive serological test, repeat serological testing showed a negative result. Clinical examination at the same follow-up visit showed complete disappearance of the frontal lesion.

Informed consent

Written informed consent was obtained from the patient for the publication of this case report and the accompanying clinical images.

## Discussion

Over the past decade, syphilis has re-emerged as a significant global public health concern. Recent estimates suggest that approximately 8 million new infections occur annually among adults aged 15-49 years, with a rising trend observed across multiple regions worldwide. This resurgence has been particularly marked among men who have sex with men (MSM), but increasing incidence has also been reported in heterosexual populations and among women of reproductive age, contributing to a worrying rise in congenital syphilis cases. Several factors have driven this epidemiological shift, including changes in sexual behaviors, gaps in screening and prevention programs, and disruptions caused by the COVID-19 pandemic, which reduced access to diagnostic and sexual health services. Furthermore, disparities in surveillance systems-especially in low- and middle-income countries-have led to underreporting, masking the true burden of the disease in many regions. In Morocco, available data remain limited, but existing national surveillance suggests that syphilis persists as an endemic infection, underscoring the need for heightened awareness among healthcare providers [3].

Beyond its clinical relevance, this case highlights the pivotal role of dental practitioners in the early recognition of systemic diseases [4]. Syphilis, a sexually transmitted infection caused by *Treponema pallidum*, remains a true “great imitator” due to its broad spectrum of clinical manifestations. Although genital lesions are the most common, uncommon extragenital presentations - particularly on the face - may easily be overlooked or misdiagnosed, leading to delays in appropriate management [1,2]. When syphilis is suspected, particularly in the presence of an atypical ulcerative lesion, clinicians should obtain a targeted, confidential, and non-judgmental sexual history. This should include recent sexual exposure, number of partners, condom use, previous sexually transmitted infections, and the presence of genital, oral, or cutaneous lesions in the patient or partners. Appropriate referral for serological confirmation and screening for associated sexually transmitted infections should also be considered. In the present case, HIV testing was recommended because of the possibility of co-infection; however, the patient declined further testing. In addition to cutaneous involvement, syphilis can also present with a variety of oral and perioral manifestations depending on the stage of infection. Primary syphilis may produce indurated, painless ulcers on the lips, tongue, buccal mucosa, or gingiva, while secondary syphilis often features mucous patches, split-papules at the commissures, or erythematous “snail-track” lesions. Tertiary syphilis, although uncommon today, may produce gummatous lesions (syphilitic gummas) involving the palate, tongue, or facial tissues, leading to destructive ulcerations and significant functional impairment if left untreated. These polymorphic oral findings can mimic traumatic, infectious, or inflammatory conditions, further complicating the diagnosis and emphasizing the importance of a thorough examination [5]. In this case, the incidental discovery of a chancre on the frontal region during routine extraoral examination underscores the importance of maintaining a comprehensive and methodical clinical approach [6]. While dentists are primarily trained to focus on the oral cavity, their field of observation naturally extends to the facial and cervical areas. The extraoral examination, sometimes considered secondary, is in fact an essential step for identifying signs of systemic, infectious, metabolic, or neoplastic disease that may otherwise go unnoticed [7].

Dentists occupy a unique position within the healthcare system, as they frequently encounter patients for preventive or routine care, often more regularly than physicians. For some individuals, the dental setting may represent their only contact with a healthcare professional [8]. Consequently, dental practitioners have repeated opportunities to recognize early manifestations of systemic disease. Several studies have emphasized the potential of dental professionals to detect signs of conditions such as diabetes, hematologic and autoimmune disorders, nutritional deficiencies, and infectious diseases, including syphilis and HIV, and to refer patients for confirmatory testing and appropriate medical management [9-11]. In terms of management, primary syphilis is treated according to well-established antibiotic regimens, with early therapy strongly associated with improved clinical outcomes and reduced transmission. Current national recommendations (Haute Autorité de Santé or HAS 2025) identify intramuscular benzathine benzylpenicillin G (2.4 million units in a single dose) as the first-line treatment for all early forms of syphilis, including primary, secondary, and early latent infections [12]. The single-dose regimen offers a major advantage in terms of adherence, facilitating complete and effective treatment. Early therapy is essential not only to ensure rapid clinical resolution but also to prevent progression to secondary or tertiary stages, reduce the risk of neurosyphilis, and interrupt onward transmission through timely partner notification and management [12]. Specifically, regarding syphilis, numerous reports and case series have documented that oral or perioral lesions may be the initial manifestation of the disease. Dental professionals are therefore in a strategic position to suspect the diagnosis and initiate timely referral for serological testing [6]. Nevertheless, gaps in knowledge and awareness of the oral and extraoral manifestations of syphilis have been reported among dental practitioners, highlighting the need for continuous education and the establishment of clear referral pathways [11].

Taken together, these observations argue for a systematic and thorough extraoral and intraoral examination during every dental consultation, coupled with a high index of suspicion when facing atypical lesions. Beyond its implications for oral health, such vigilance contributes to the early detection and management of systemic diseases, representing a valuable contribution of dentistry to overall public health. This case thus serves as a reminder that clinical rigor, keen observation, and diagnostic curiosity remain essential attributes in daily dental practice.

## Conclusions

This case underscores the pivotal role of dental practitioners in the early detection of systemic diseases through meticulous extraoral examination. The incidental identification of a primary syphilitic lesion during a routine dental visit highlights how comprehensive clinical assessment extends beyond oral structures to encompass general health surveillance. Reinforcing awareness and training regarding the atypical presentations of infectious diseases such as syphilis can enhance timely diagnosis and interdisciplinary referral, ultimately improving patient outcomes and strengthening the contribution of dentistry to public health.
